# Supplementary material for: Transcriptomic Analysis of Four Cerianthid (Cnidaria, Ceriantharia) Venoms
Source: Mar Drugs. 2020 Aug 5;18(8):413. doi: 10.3390/md18080413 (PMC7460484; doi:10.3390/md18080413)
Supplement: Supplementary file 1 [file marinedrugs-18-00413-s001.zip › supplementary-figures.pdf]

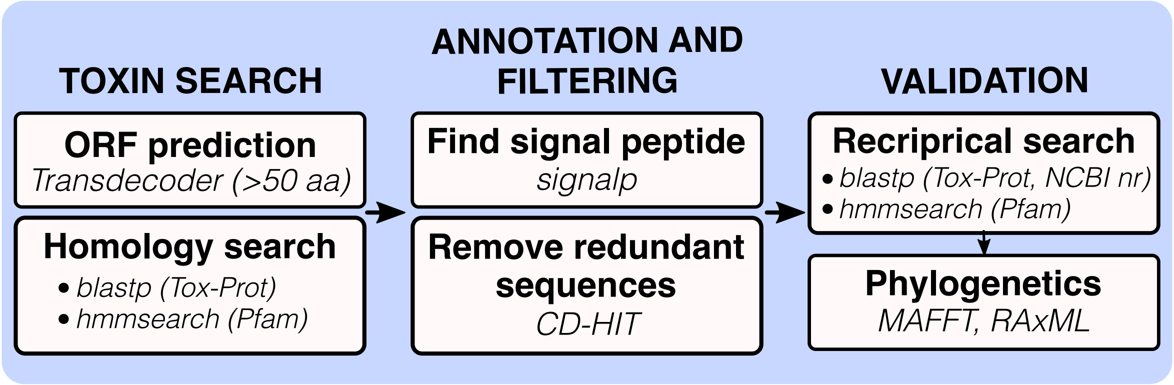

Figure S1. Bioinformatic pipeline for the annotation of venom-like genes for four cerianthid transcriptomes.

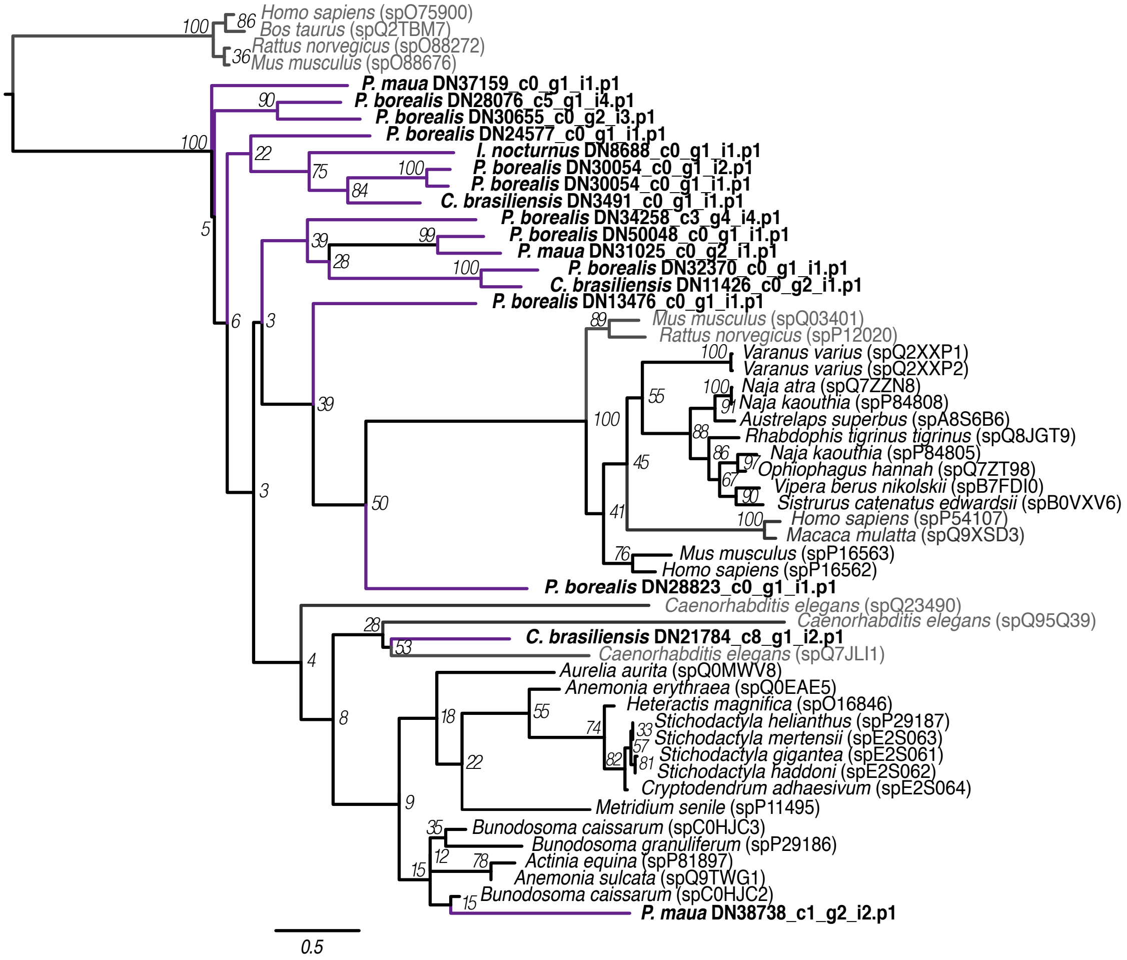

Figure S2. Phylogenetic gene tree of cystine-rich protein family sequences. The cysteine-rich protein gene tree was constructed using RAxML with the WAG+G model. Bootstrap support based on 500 rapid bootstrap replicates, and all support values are shown. Sequence names in bold with purple branches are those from cerianthids. Sequences in gray are non-venomous representatives. Phylogeny modified from [81].

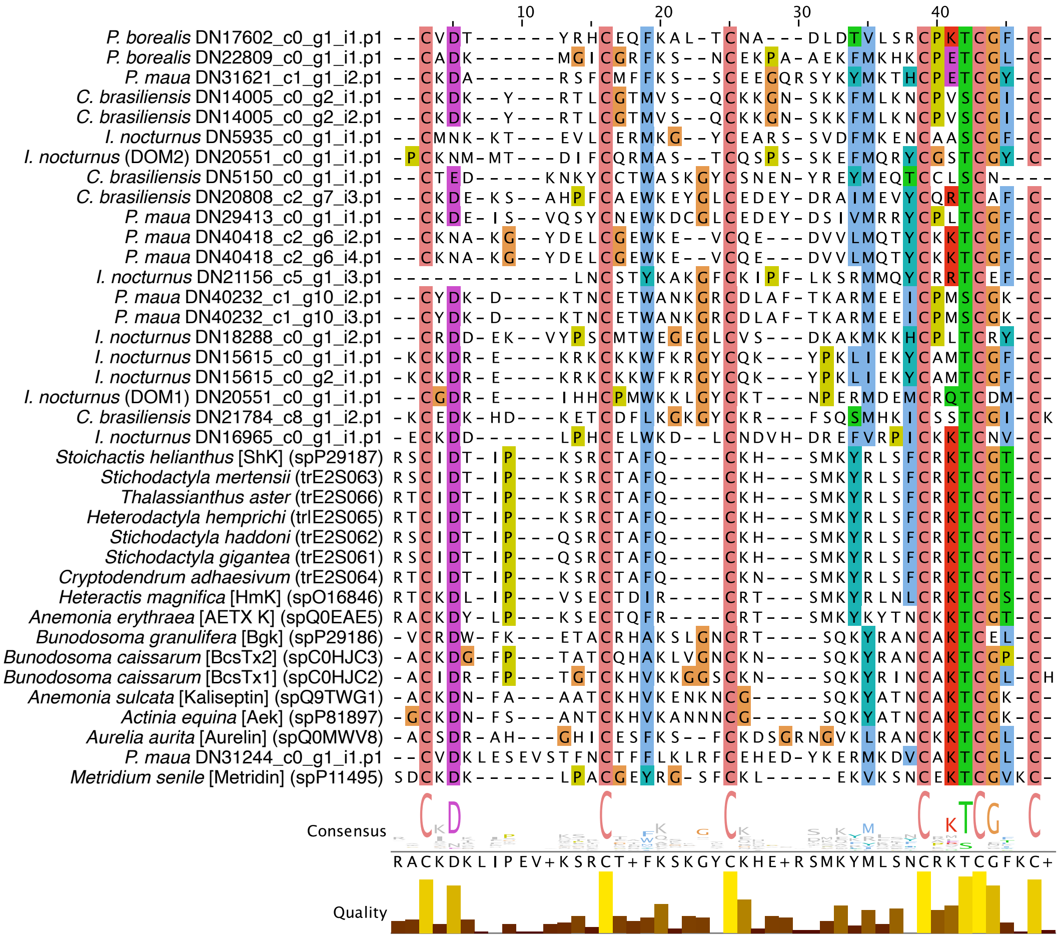

Figure S3. Multiple sequence alignment of ShK domains in ceriantid venom-like genes and other toxin representatives. Sequences alignment constructed using the L-INS-I algorithm in MAFFT [153] and viewed in a Clustal color scheme using Jalview v2.11.1 [155]. Six conserved cystine residues characteristic of ShK domains are should across all taxa. Various SHK toxin domains modified from [81].

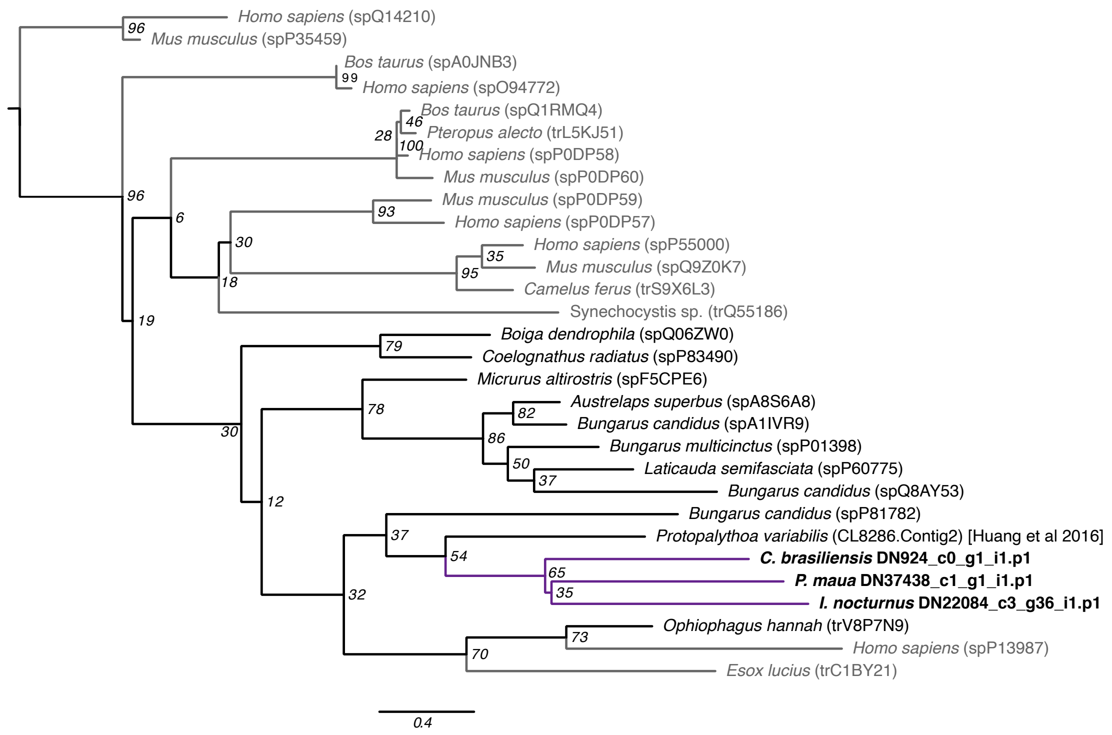

Figure S4. Phylogenetic gene tree of three-finger toxin family sequences. The three-finger gene tree was constructed using RAxML with the the WAG+I+G model. Bootstrap support based on 500 rapid bootstrap replicates, and all support values are shown. Sequence names in bold with purple branches are those from cerianthids. Sequences in gray are non-venomous representatives. Phylogeny modified from [47].

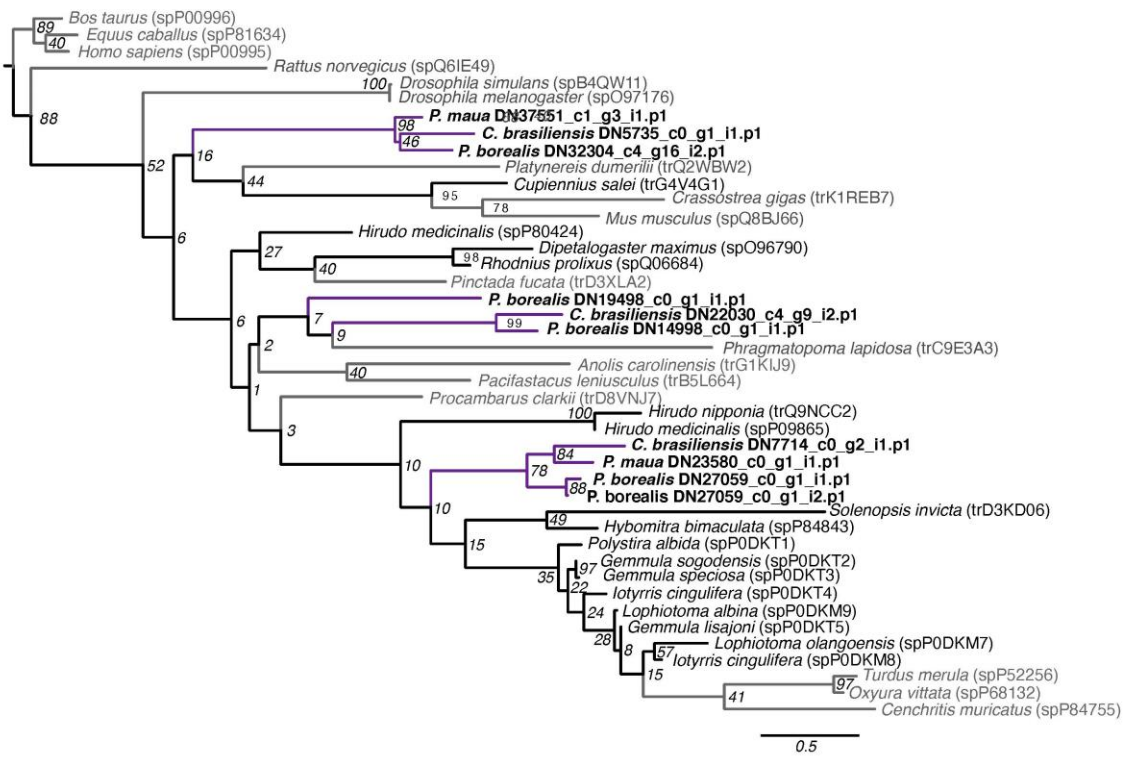

Figure S5. Phylogenetic gene tree of kazal family sequences. The kazal gene tree was constructed using RAxML with the WAG+I+G model. Bootstrap support based on 500 rapid bootstrap replicates, and all support values are shown. Sequence names in bold with purple branches are those from cerianthids. Sequences in gray are non-venomous representatives. Phylogeny modified from [81].

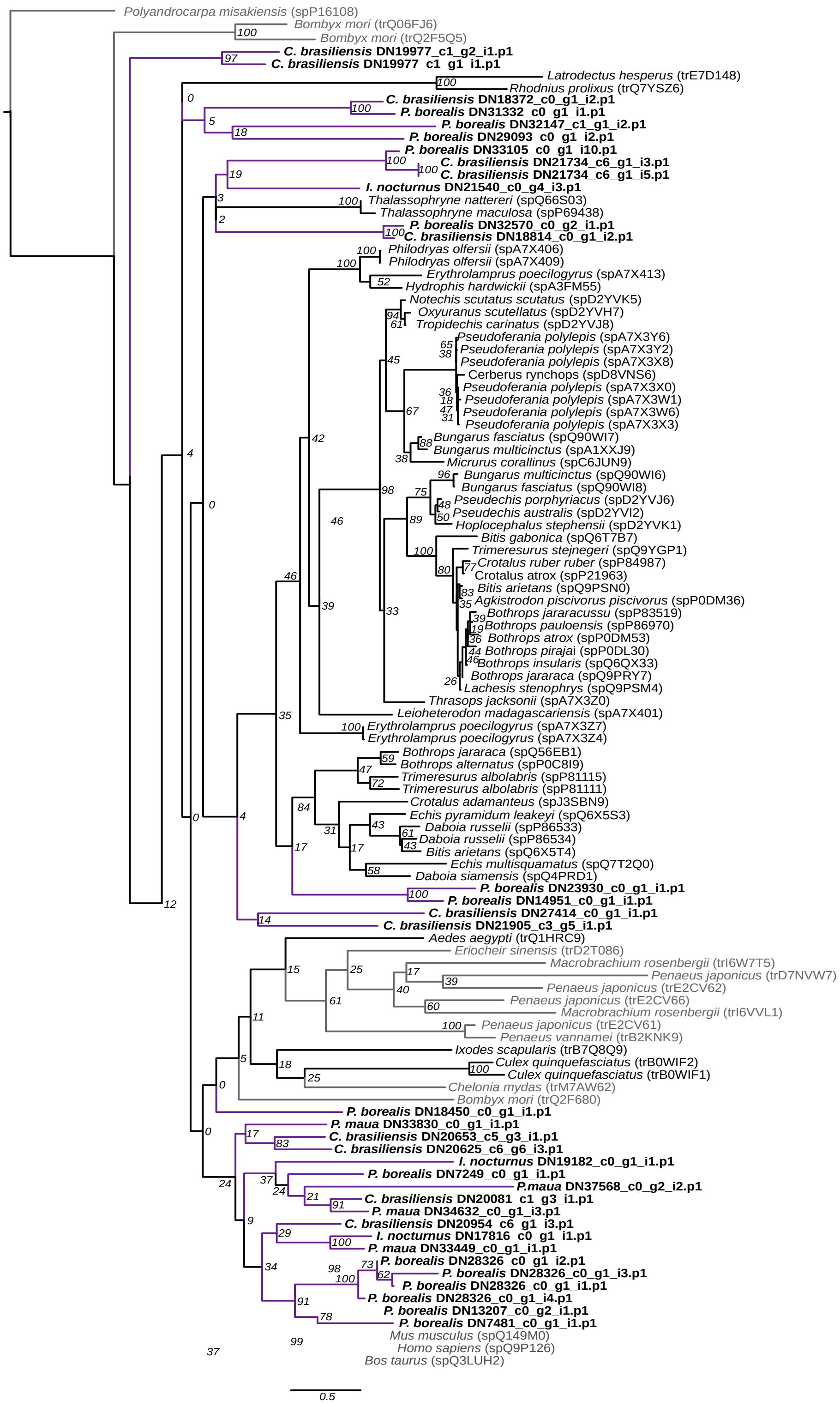

Figure S6. Phylogenetic gene tree of C-type lectin family sequences. The C-type lectin gene tree was constructed using RAxML with the WAG+I+G model. Bootstrap support based on 500 rapid bootstrap replicates, and all support values are shown. Sequence names in bold with purple branches are those from cerianthids. Sequences in gray are non-venomous representatives. Phylogeny modified from [81].

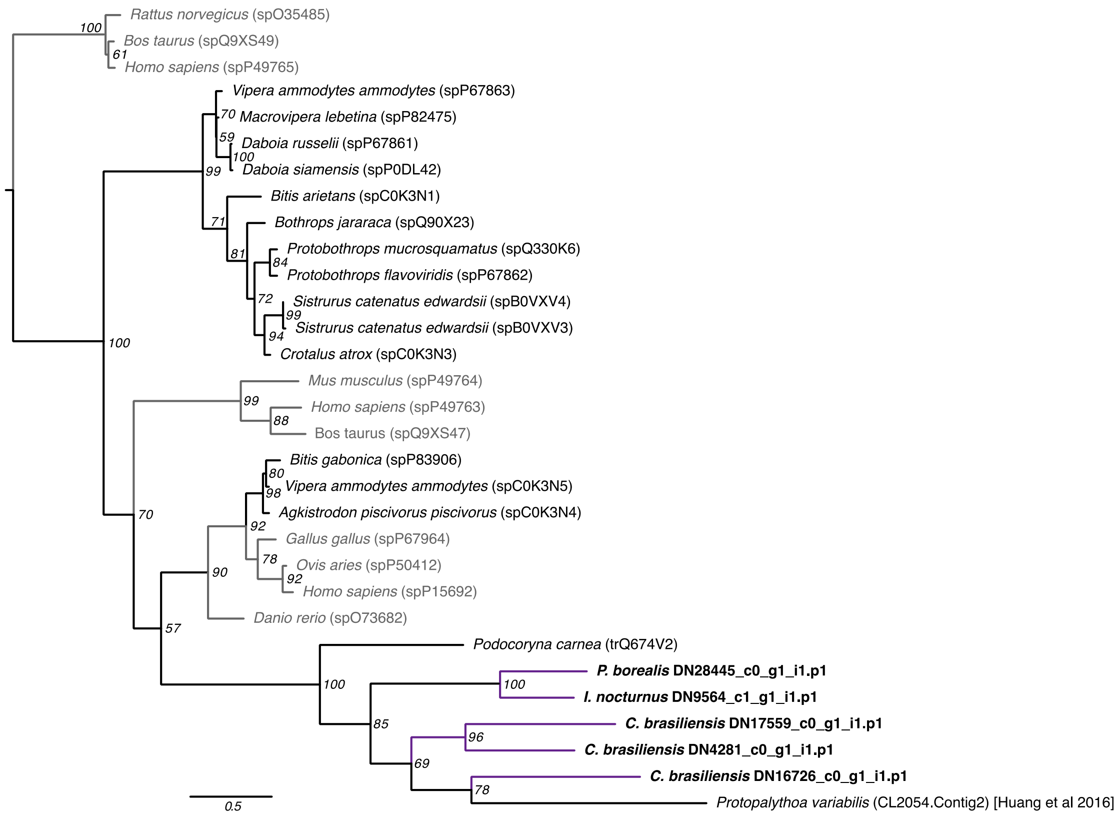

Figure S7. Phylogenetic gene tree of venom vascular endothelial growth factors (venom VEGF) family sequences. The venom VEGF gene tree was constructed using RAxML with the WAG+G model. Bootstrap support based on 500 rapid bootstrap replicates, and all support values are shown. Sequence names in bold with purple branches are those from cerianthids. Sequences in gray are non-venomous representatives. Phylogeny modified from [47].

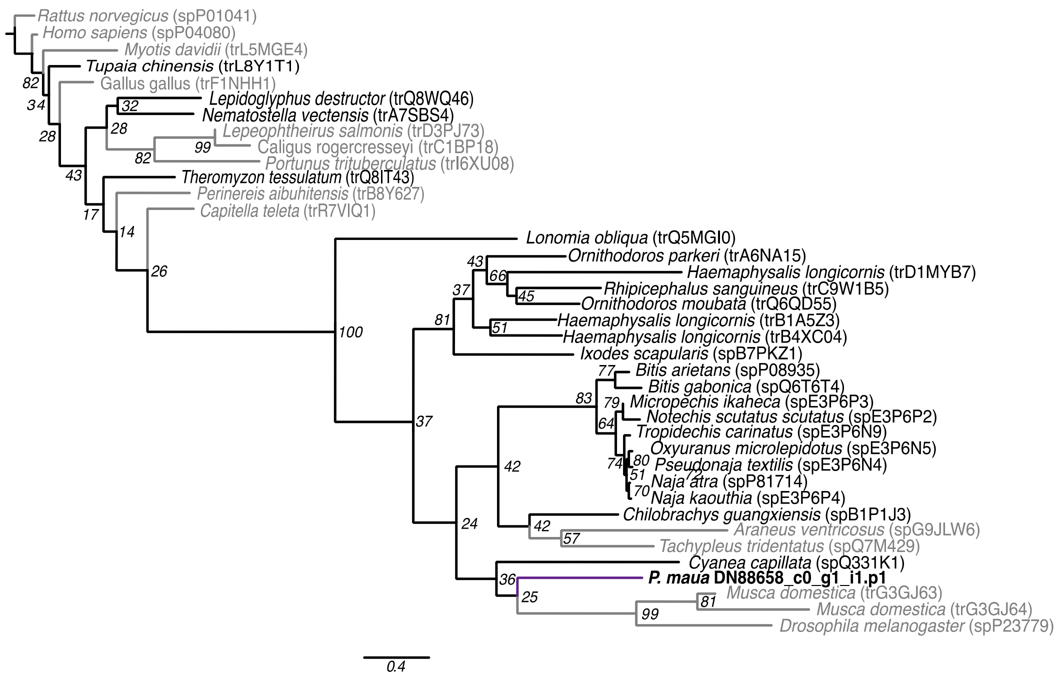

Figure S8. Phylogenetic gene tree of cystatin family sequences. The cystatin gene tree was constructed using RAxML with the WAG+I+G model. Bootstrap support based on 500 rapid bootstrap replicates, and all support values are shown. Sequence names in bold with purple branches are those from cerianthids. Sequences in gray are non-venomous representatives. Phylogeny modified from [81].

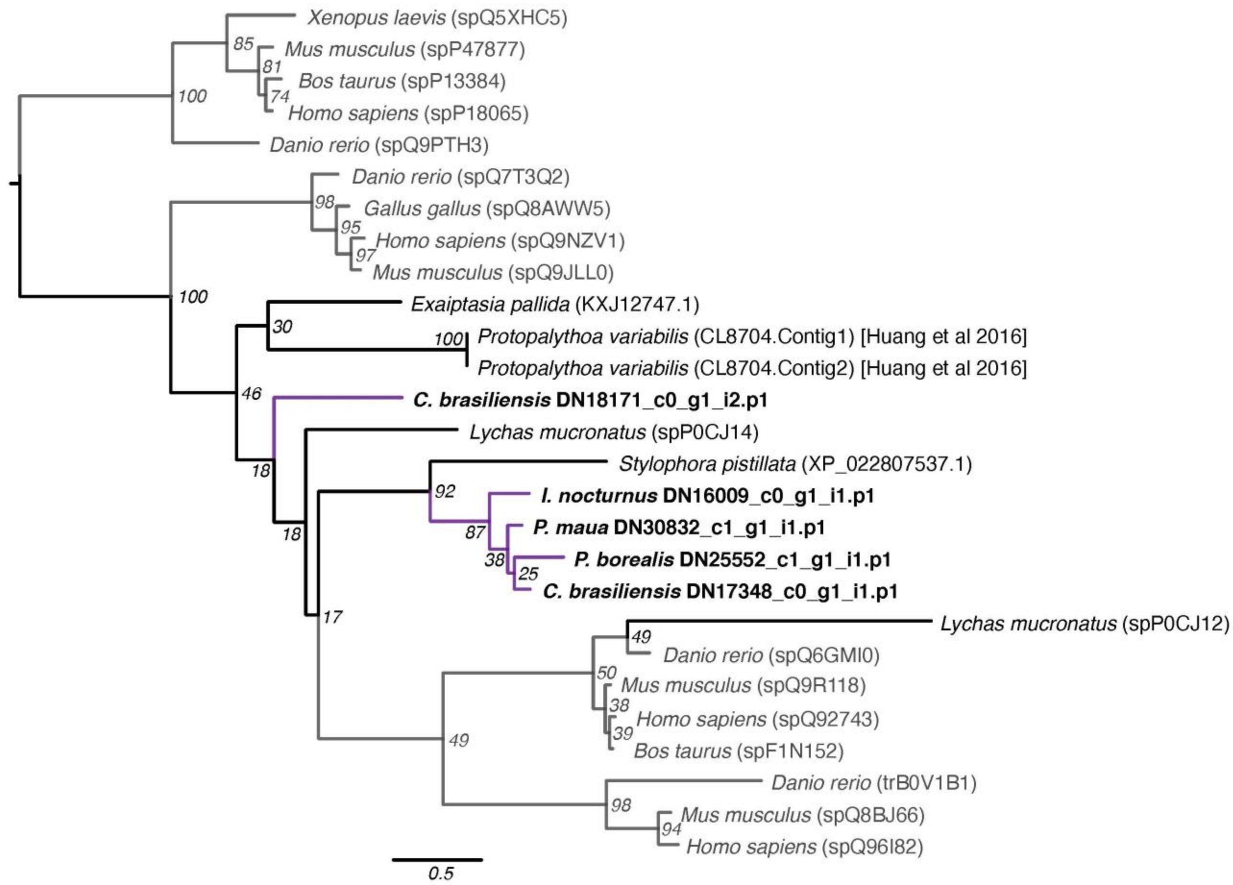

Figure S9. Phylogenetic gene tree of venom protein 302 family sequences. The venom protein 302 gene tree was constructed using RAxML with the JTT+I+G model. Bootstrap support based on 500 rapid bootstrap replicates, and all support values are shown. Sequence names in bold with purple branches are those from cerianthids. Sequences in gray are non-venomous representatives. Phylogeny modified from [47].

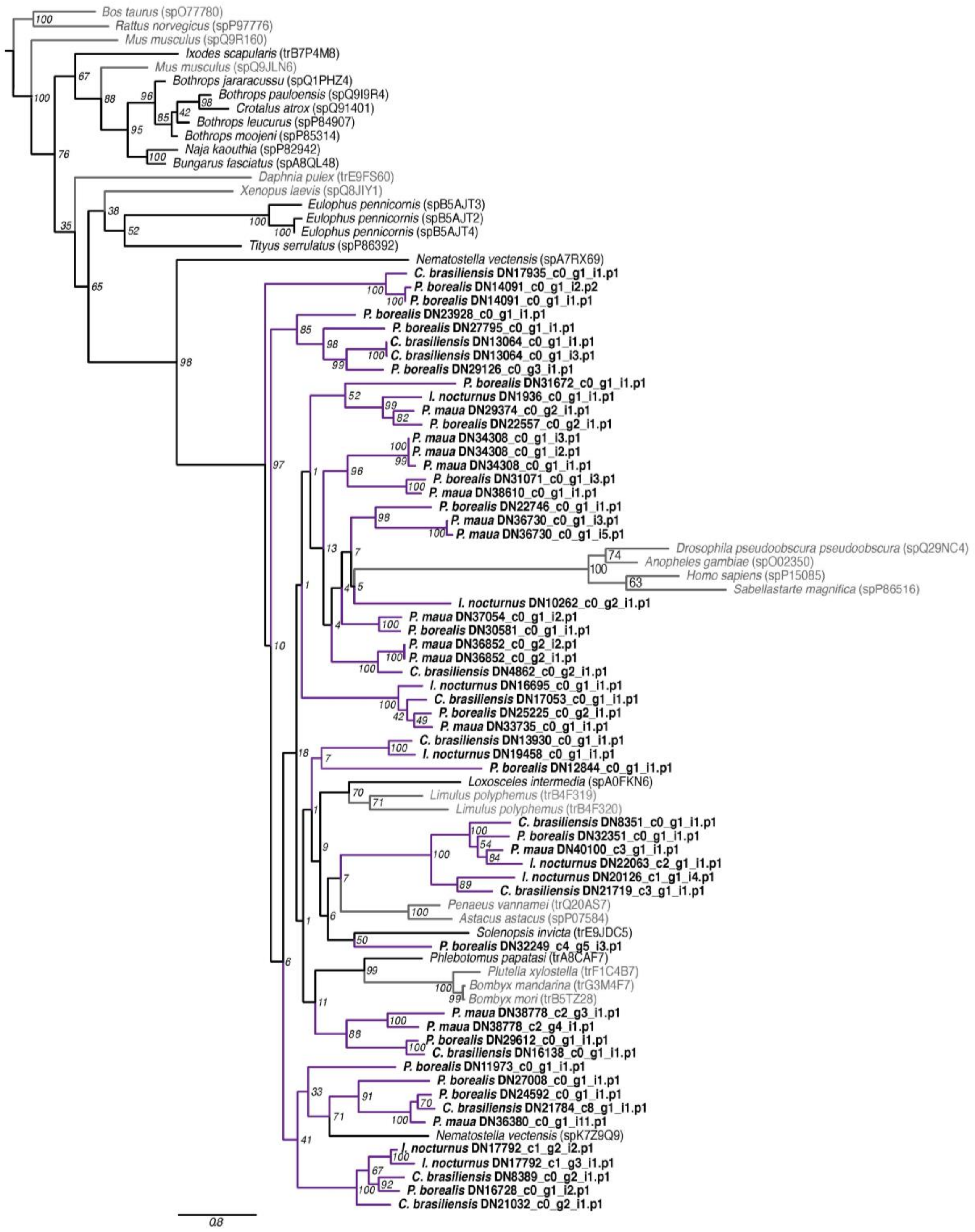

Figure S10. Phylogenetic gene tree of metalloprotease M12A family sequences. The metalloprotease M12A gene tree was constructed using RAxML with the WAG+I+G model. Bootstrap support based on 500 rapid bootstrap replicates, and all support values are shown. Sequence names in bold with purple branches are those from cerianthids. Sequences in gray are non-venomous representatives. Phylogeny modified from [81].

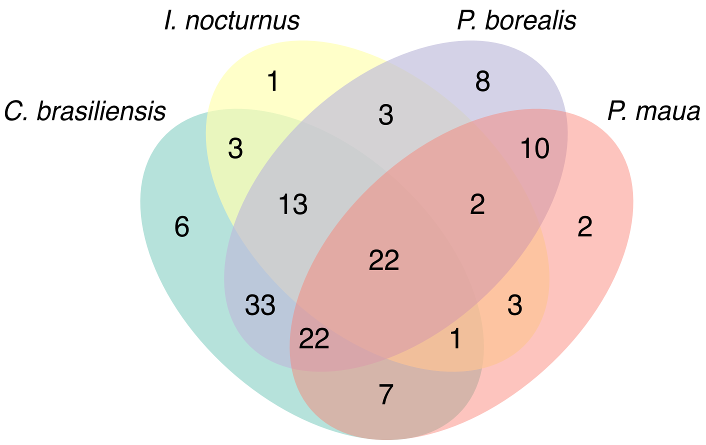

Species	# of proteins, (in cluster)	# of proteins, (singletons)
C. brasiliensis	107	27
I. nocturnus	48	13
P. borealis	113	43
P. maua.	69	18

Figure S11. Orthologous gene clusters of the putative venom-like genes for all four cerianthids. Venn diagram shows overlap of gene clusters for all four species. Table shows the number of venom-like genes within each species that fall within a cluster or are singletons, which as genes that cannot be placed into a cluster (unique to each species). Gene clusters were analyzed using the OrthoVenn2 server under default setting except evalue changed to 1e-5 (https://orthovenn2.bioinfotoolkits.net/) [157].

Supplemental References

[157] Xu, L.; Dong, Z.; Fang, L.; Luo, Y.; Wei, Z.; Guo, H.; Zhang, G.; Gu, Y.Q.; Coleman-Derr, D.; Xia, Q.; et al. OrthoVenn2: A web server for whole-genome comparison and annotation of orthologous clusters across multiple species. Nucleic Acids Res. 201
