# Supplementary material for: Transcriptomic Analysis of Four Cerianthid (Cnidaria, Ceriantharia) Venoms
Source: Mar Drugs. 2020 Aug 5;18(8):413. doi: 10.3390/md18080413 (PMC7460484; doi:10.3390/md18080413)
Supplement: Supplementary file 1 [file marinedrugs-18-00413-s001.zip › Individual supplementary_figs.zip!/supplementary_figs/SuppFig1_annot_pipeline.pdf]

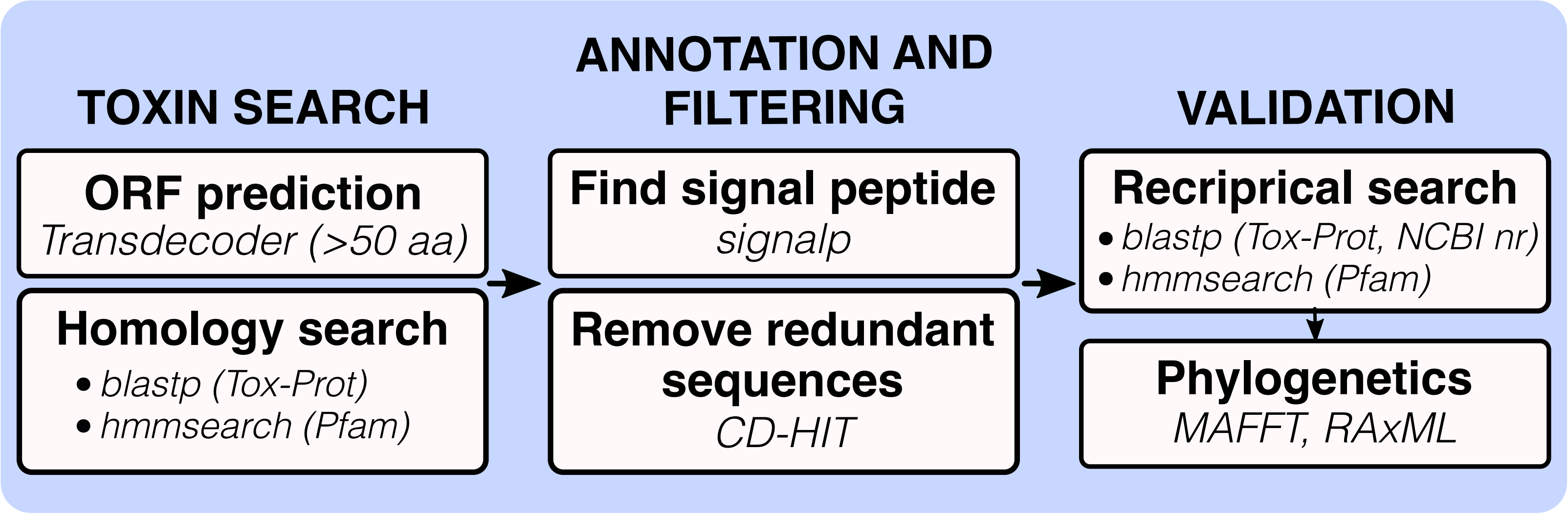
TOXIN SEARCH
ORF prediction
Transdecoder (>50 aa)
Homology search
• blastp (Tox-Prot)
• hmmsearch (Pfam)
ANNOTATION AND FILTERING
Find signal peptide
signalp
Remove redundant sequences
CD-HIT
VALIDATION
Recriprical search
• blastp (Tox-Prot, NCBI nr)
• hmmsearch (Pfam)
Phylogenetics
MAFFT, RAxML
