# Supplementary material for: Transcriptomic Analysis of Four Cerianthid (Cnidaria, Ceriantharia) Venoms
Source: Mar Drugs. 2020 Aug 5;18(8):413. doi: 10.3390/md18080413 (PMC7460484; doi:10.3390/md18080413)
Supplement: Supplementary file 1 [file marinedrugs-18-00413-s001.zip › Individual supplementary_figs.zip!/supplementary_figs/SuppFig10_orthovenn.pdf]

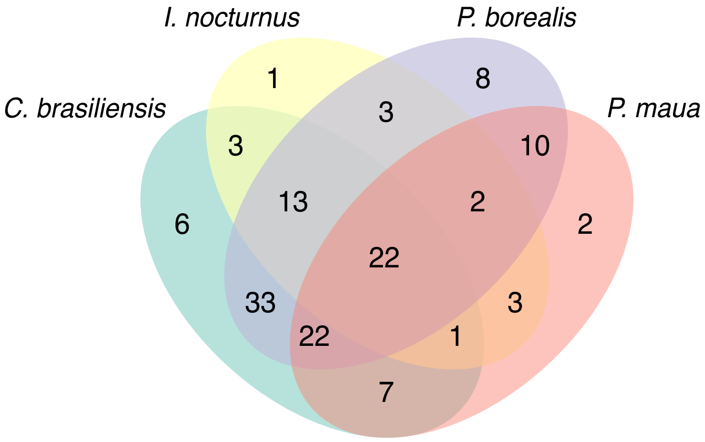

Species	# of proteins, (in cluster)	# of proteins, (singletons)
C. brasiliensis	107	27
I. nocturnus	48	13
P. borealis	113	43
P. maua.	69	18
