# Supplementary material for: Transcriptomic Analysis of Four Cerianthid (Cnidaria, Ceriantharia) Venoms
Source: Mar Drugs. 2020 Aug 5;18(8):413. doi: 10.3390/md18080413 (PMC7460484; doi:10.3390/md18080413)
Supplement: Supplementary file 1 [file marinedrugs-18-00413-s001.zip › Individual supplementary_figs.zip!/supplementary_figs/SuppFig2_cerianthid_CRP_.pdf]

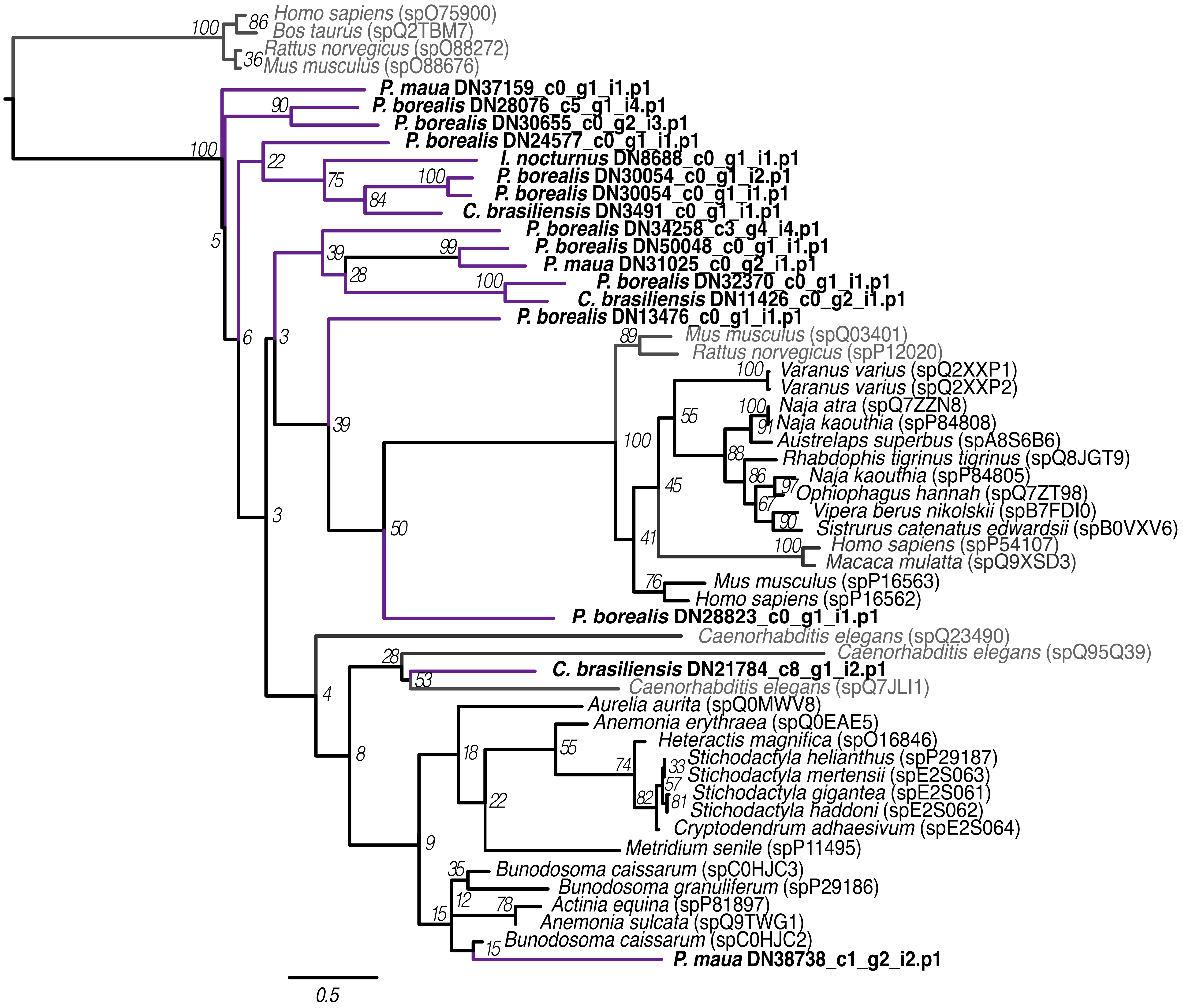
Homo sapiens (spO75900)
Bos taurus (spQ2TBM7)
Rattus norvegicus (spO88272)
Mus musculus (spO88676)
P. maua DN37159_c0_g1_i1.p1
P. borealis DN28076_c5_g1_i4.p1
P. borealis DN30655_c0_g2_i3.p1
P. borealis DN24577_c0_g1_i1.p1
I. nocturnus DN8688_c0_g1_i1.p1
P. borealis DN30054_c0_g1_i2.p1
P. borealis DN30054_c0_g1_i1.p1
C. brasiliensis DN3491_c0_g1_i1.p1
P. borealis DN34258_c3_g4_i4.p1
P. borealis DN50048_c0_g1_i1.p1
P. maua DN31025_c0_g2_i1.p1
P. borealis DN32370_c0_g1_i1.p1
C. brasiliensis DN11426_c0_g2_i1.p1
P. borealis DN13476_c0_g1_i1.p1
Mus musculus (spQ03401)
Rattus norvegicus (spP12020)
Varanus varius (spQ2XXP1)
Varanus varius (spQ2XXP2)
Naja atra (spQ7ZZN8)
Naja kaouthia (spP84808)
Austrelaps superbus (spA8S6B6)
Rhabdophis tigrinus tigrinus (spQ8JGT9)
Naja kaouthia (spP84805)
Ophiophagus hannah (spQ7ZT98)
Vipera berus nikolskii (spB7FDI0)
Sistrurus catenatus edwardsii (spB0VXV6)
Homo sapiens (spP54107)
Macaca mulatta (spQ9XSD3)
Mus musculus (spP16563)
Homo sapiens (spP16562)
P. borealis DN28823_c0_g1_i1.p1
Caenorhabditis elegans (spQ23490)
Caenorhabditis elegans (spQ95Q39)
C. brasiliensis DN21784_c8_g1_i2.p1
Caenorhabditis elegans (spQ7JLI1)
Aurelia aurita (spQ0MWV8)
Anemonia erythraea (spQ0EAE5)
Heteractis magnifica (spO16846)
Stichodactyla helianthus (spP29187)
Stichodactyla mertensii (spE2S063)
Stichodactyla gigantea (spE2S061)
Stichodactyla haddoni (spE2S062)
Cryptodendrum adhaesivum (spE2S064)
Metridium senile (spP11495)
Bunodosoma caissarum (spC0HJC3)
Bunodosoma granuliferum (spP29186)
Actinia equina (spP81897)
Anemonia sulcata (spQ9TWG1)
Bunodosoma caissarum (spC0HJC2)
P. maua DN38738_c1_g2_i2.p1
0.5
