# Supplementary material for: Transcriptomic Analysis of Four Cerianthid (Cnidaria, Ceriantharia) Venoms
Source: Mar Drugs. 2020 Aug 5;18(8):413. doi: 10.3390/md18080413 (PMC7460484; doi:10.3390/md18080413)
Supplement: Supplementary file 1 [file marinedrugs-18-00413-s001.zip › Individual supplementary_figs.zip!/supplementary_figs/SuppFig3_shk.pdf]

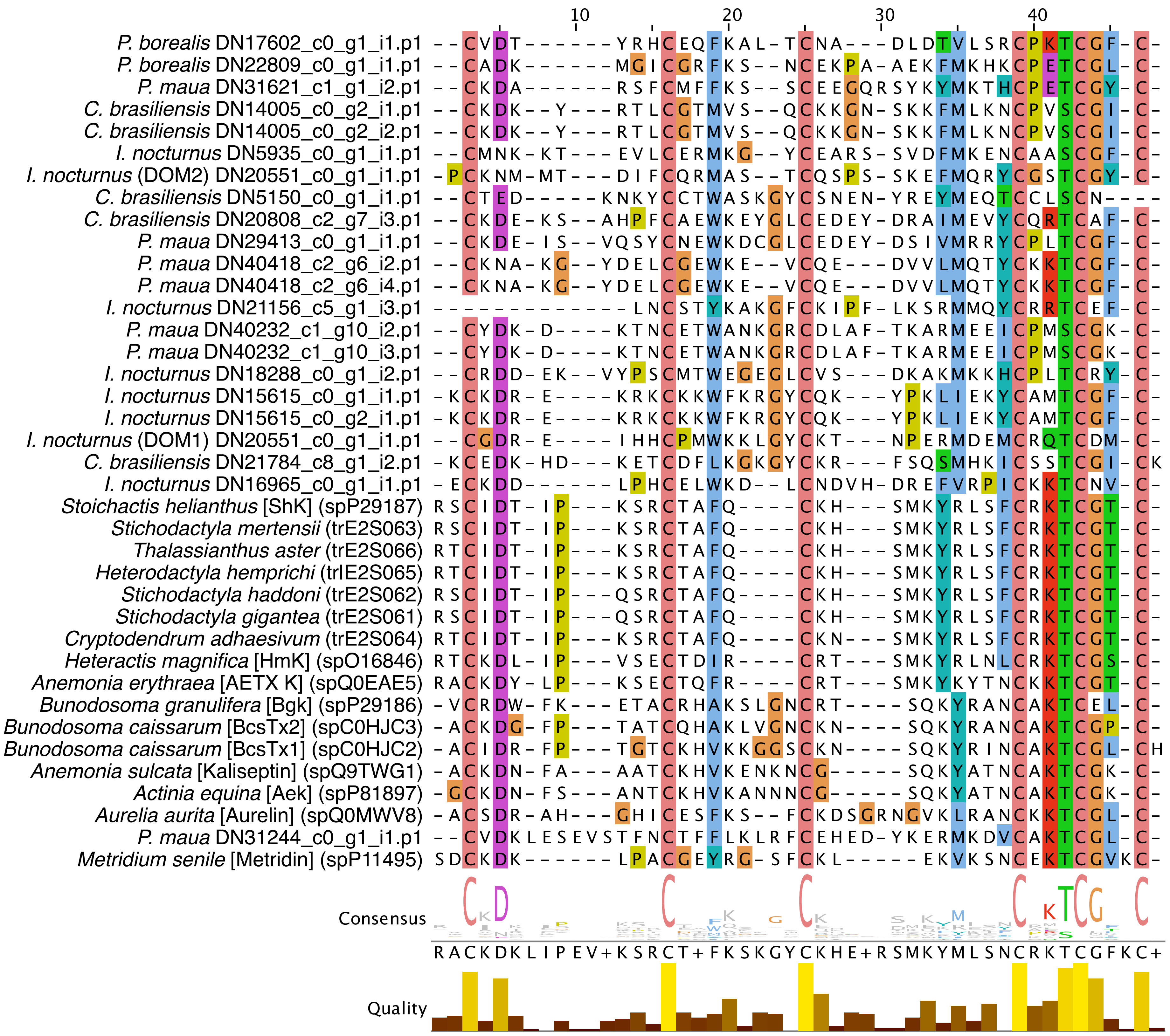
10
20
30
40
P. borealis DN17602_c0_g1_i1.p1 --CVDT------YRHCEQFKAL-TCNA---DLDTVLSRCPKTCGF-C-
P. borealis DN22809_c0_g1_i1.p1 --CADK------MGICGRFKS--NCEKPA-AEKFMKHKCPETCGL-C-
P. maua DN31621_c1_g1_i2.p1 --CKDA------RSFCMFFKS--SCEEGQRSYKYMKTHCPETCGY-C-
C. brasiliensis DN14005_c0_g2_i1.p1 --CKDK--Y---RTLCGTMVS--QCKKGN-SKKFMLKNCPVSCGI-C-
C. brasiliensis DN14005_c0_g2_i2.p1 --CKDK--Y---RTLCGTMVS--QCKKGN-SKKFMLKNCPVSCGI-C-
I. nocturnus DN5935_c0_g1_i1.p1 --CMNK-KT---EVLCERMKG--YCEARS-SVDFMKENCAASCGF-C-
I. nocturnus (DOM2) DN20551_c0_g1_i1.p1 -PCKNM-MT---DIFCQRMAS--TCQSPS-SKEFMQRYCGSTCGY-C-
C. brasiliensis DN5150_c0_g1_i1.p1 --CTED-----KNKYCCTWASKGYCSNEN-YREYMEQTCCLSCN----
C. brasiliensis DN20808_c2_g7_i3.p1 --CKDE-KS--AHPFCAEWKEYGLCEDEY-DRAIMEVYCQRTCAF-C-
P. maua DN29413_c0_g1_i1.p1 --CKDE-IS--VQSYCNEWKDCGLCEDEY-DSIVMRRYCPLTCGF-C-
P. maua DN40418_c2_g6_i2.p1 --CKNA-KG--YDELCGEWKE--VCQE---DVVLMQTYCKKTCGF-C-
P. maua DN40418_c2_g6_i4.p1 --CKNA-KG--YDELCGEWKE--VCQE---DVVLMQTYCKKTCGF-C-
I. nocturnus DN21156_c5_g1_i3.p1 -------------LNCSTYKAKGFCKIPF-LKSRMMQYCRRTCEF-C-
P. maua DN40232_c1_g10_i2.p1 --CYDK-D----KTNCETWANKGRCDLAF-TKARMEEICPMSCGK-C-
P. maua DN40232_c1_g10_i3.p1 --CYDK-D----KTNCETWANKGRCDLAF-TKARMEEICPMSCGK-C-
I. nocturnus DN18288_c0_g1_i2.p1 --CRDD-EK--VYPSCMTWEGEGLCVS---DKAKMKKHCPLTCRY-C-
I. nocturnus DN15615_c0_g1_i1.p1 -KCKDR-E----KRKCKKWFKRGYCQK---YPKLIEKYCAMTCGF-C-
I. nocturnus DN15615_c0_g2_i1.p1 -KCKDR-E----KRKCKKWFKRGYCQK---YPKLIEKYCAMTCGF-C-
I. nocturnus (DOM1) DN20551_c0_g1_i1.p1 --CGDR-E----IHHCPMWKKLGYCKT---NPERMDEMCRQTCDM-C-
C. brasiliensis DN21784_c8_g1_i2.p1 -KCEDK-HD---KETCDFLKGKGYCKR---FSQSMHKICSSTCGI-CK
I. nocturnus DN16965_c0_g1_i1.p1 -ECKDD------LPHCELWKD--LCNDVH-DREFVRPICKKTCNV-C-
Stoichactis helianthus [ShK] (spP29187) RSCIDT-IP---KSRCTAFQ----CKH---SMKYRLSFCRKTCGT-C-
Stichodactyla mertensii (trE2S063) RSCIDT-IP---KSRCTAFQ----CKH---SMKYRLSFCRKTCGT-C-
Thalassianthus aster (trE2S066) RTCIDT-IP---KSRCTAFQ----CKH---SMKYRLSFCRKTCGT-C-
Heterodactyla hemprichi (trlE2S065) RTCIDT-IP---KSRCTAFQ----CKH---SMKYRLSFCRKTCGT-C-
Stichodactyla haddoni (trE2S062) RSCIDT-IP---QSRCTAFQ----CKH---SMKYRLSFCRKTCGT-C-
Stichodactyla gigantea (trE2S061) RSCIDT-IP---QSRCTAFQ----CKH---SMKYRLSFCRKTCGT-C-
Cryptodendrum adhaesivum (trE2S064) RTCIDT-IP---KSRCTAFQ----CKN---SMKYRLSFCRKTCGT-C-
Heteractis magnifica [HmK] (spO16846) RTCKDL-IP---VSECTDIR----CRT---SMKYRLNLCRKTCGS-C-
Anemonia erythraea [AETX K] (spQ0EAE5) RACKDY-LP---KSECTQFR----CRT---SMKYKYTNCKKTCGT-C-
Bunodosoma granulifera [Bgk] (spP29186) -VCRDW-FK---ETACRHAKSLGNCRT----SQKYRANCAKTCEL-C-
Bunodosoma caissarum [BcsTx2] (spC0HJC3) -ACKDG-FP---TATCQHAKLVGNCKN----SQKYRANCAKTCGP-C-
Bunodosoma caissarum [BcsTx1] (spC0HJC2) -ACIDR-FP---TGTCKHVKKGGSCKN----SQKYRINCAKTCGL-CH
Anemonia sulcata [Kaliseptin] (spQ9TWG1) -ACKDN-FA---AATCKHVKENKNCG-----SQKYATNCAKTCGK-C-
Actinia equina [Aek] (spP81897) -GCKDN-FS---ANTCKHVKANNNCG-----SQKYATNCAKTCGK-C-
Aurelia aurita [Aurelin] (spQ0MWV8) -ACSDR-AH---GHICESFKS--FCKDSGRNGVKLRANCKKTCGL-C-
P. maua DN31244_c0_g1_i1.p1 --CVDKLESEVSTFNCTFFLKLRFCEHED-YKERMKDVCAKTCGL-C-
Metridium senile [Metridin] (spP11495) SDCKDK------LPACGEYRG-SFCKL-----EKVKSNCEKTCGVKC-
Consensus
RACKDKLIPEV+KSRCT+FKSKGYCKHE+RSMKYMLSNCRKTCGFKC+
Quality
