# Supplementary material for: Transcriptomic Analysis of Four Cerianthid (Cnidaria, Ceriantharia) Venoms
Source: Mar Drugs. 2020 Aug 5;18(8):413. doi: 10.3390/md18080413 (PMC7460484; doi:10.3390/md18080413)
Supplement: Supplementary file 1 [file marinedrugs-18-00413-s001.zip › Individual supplementary_figs.zip!/supplementary_figs/SuppFig4_cerianthid_TFT.pdf]

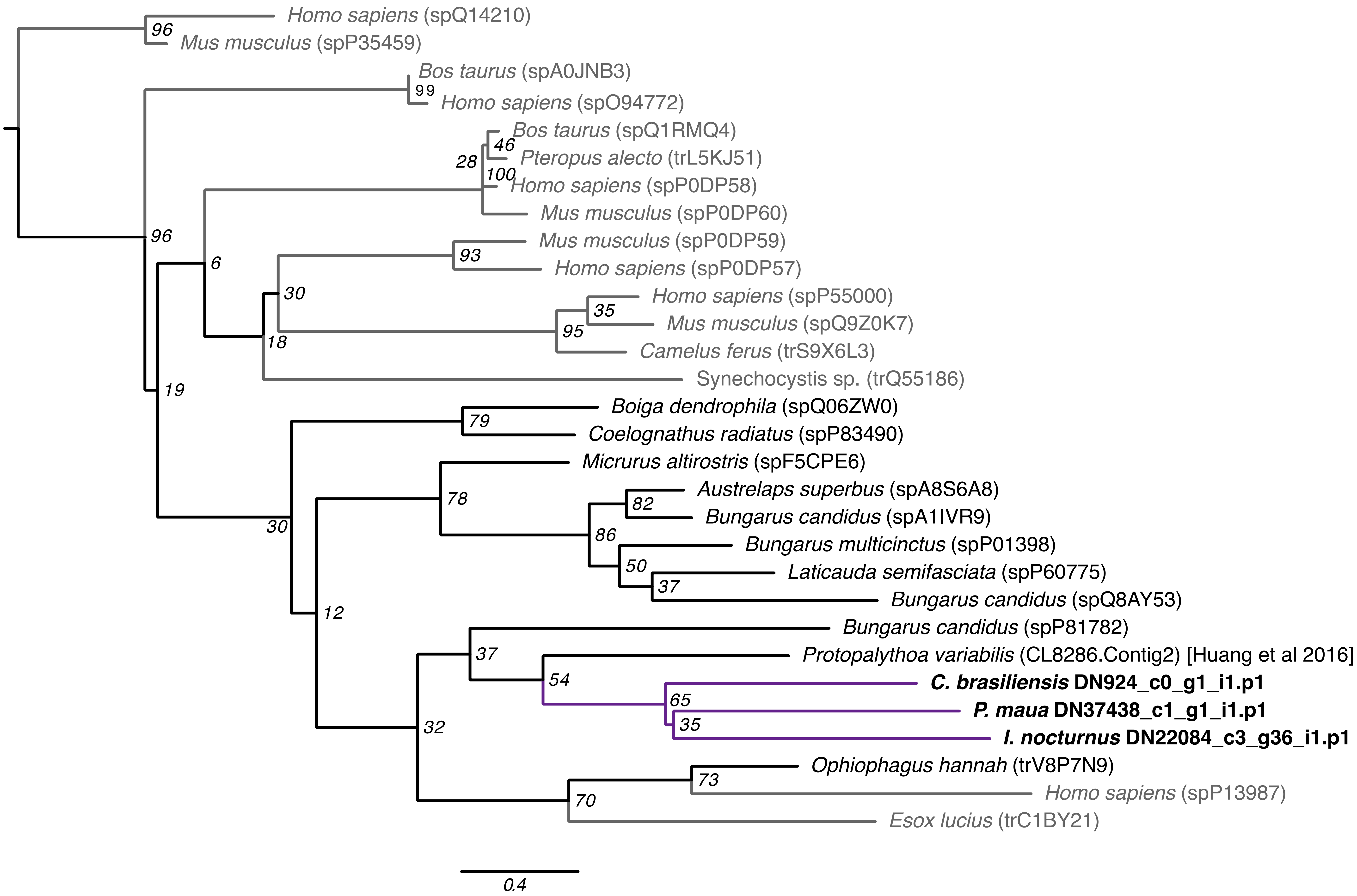
Homo sapiens (spQ14210)
Mus musculus (spP35459)
Bos taurus (spA0JNB3)
Homo sapiens (spO94772)
Bos taurus (spQ1RMQ4)
Pteropus alecto (trL5KJ51)
Homo sapiens (spP0DP58)
Mus musculus (spP0DP60)
Mus musculus (spP0DP59)
Homo sapiens (spP0DP57)
Homo sapiens (spP55000)
Mus musculus (spQ9Z0K7)
Camelus ferus (trS9X6L3)
Synechocystis sp. (trQ55186)
Boiga dendrophila (spQ06ZW0)
Coelognathus radiatus (spP83490)
Micrurus altirostris (spF5CPE6)
Austrelaps superbus (spA8S6A8)
Bungarus candidus (spA1IVR9)
Bungarus multicinctus (spP01398)
Laticauda semifasciata (spP60775)
Bungarus candidus (spQ8AY53)
Bungarus candidus (spP81782)
Protopalythoa variabilis (CL8286.Contig2) [Huang et al 2016]
C. brasiliensis DN924_c0_g1_i1.p1
P. maua DN37438_c1_g1_i1.p1
I. nocturnus DN22084_c3_g36_i1.p1
Ophiophagus hannah (trV8P7N9)
Homo sapiens (spP13987)
Esox lucius (trC1BY21)
96
99
46
28
100
96
6
93
30
35
95
18
19
79
78
82
86
50
37
30
12
37
54
65
35
32
73
70
0.4
