# Supplementary material for: Transcriptomic Analysis of Four Cerianthid (Cnidaria, Ceriantharia) Venoms
Source: Mar Drugs. 2020 Aug 5;18(8):413. doi: 10.3390/md18080413 (PMC7460484; doi:10.3390/md18080413)
Supplement: Supplementary file 1 [file marinedrugs-18-00413-s001.zip › Individual supplementary_figs.zip!/supplementary_figs/SuppFig5_cerianthid_kazal.pdf]

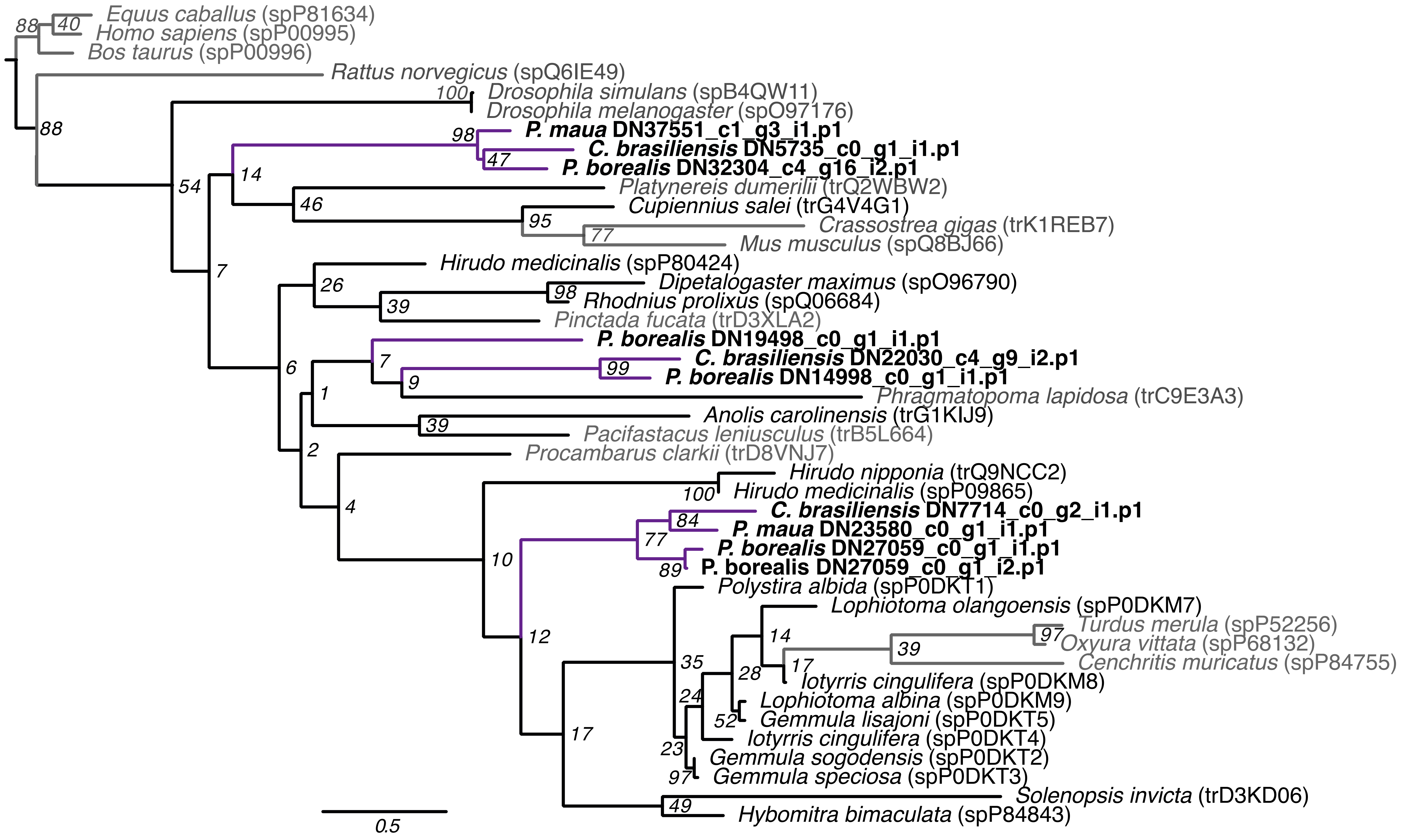
Equus caballus (spP81634)
Homo sapiens (spP00995)
Bos taurus (spP00996)
Rattus norvegicus (spQ6IE49)
Drosophila simulans (spB4QW11)
Drosophila melanogaster (spO97176)
P. maua DN37551_c1_g3_i1.p1
C. brasiliensis DN5735_c0_g1_i1.p1
P. borealis DN32304_c4_g16_i2.p1
Platynereis dumerilii (trQ2WBW2)
Cupiennius salei (trG4V4G1)
Crassostrea gigas (trK1REB7)
Mus musculus (spQ8BJ66)
Hirudo medicinalis (spP80424)
Dipetalogaster maximus (spO96790)
Rhodnius prolixus (spQ06684)
Pinctada fucata (trD3XLA2)
P. borealis DN19498_c0_g1_i1.p1
C. brasiliensis DN22030_c4_g9_i2.p1
P. borealis DN14998_c0_g1_i1.p1
Phragmatopoma lapidosa (trC9E3A3)
Anolis carolinensis (trG1KIJ9)
Pacifastacus leniusculus (trB5L664)
Procambarus clarkii (trD8VNJ7)
Hirudo nipponia (trQ9NCC2)
Hirudo medicinalis (spP09865)
C. brasiliensis DN7714_c0_g2_i1.p1
P. maua DN23580_c0_g1_i1.p1
P. borealis DN27059_c0_g1_i1.p1
P. borealis DN27059_c0_g1_i2.p1
Polystira albida (spP0DKT1)
Lophiotoma olangoensis (spP0DKM7)
Turdus merula (spP52256)
Oxyura vittata (spP68132)
Cenchritis muricatus (spP84755)
Iotyrris cingulifera (spP0DKM8)
Lophiotoma albina (spP0DKM9)
Gemmula lisajoni (spP0DKT5)
Iotyrris cingulifera (spP0DKT4)
Gemmula sogodensis (spP0DKT2)
Gemmula speciosa (spP0DKT3)
Solenopsis invicta (trD3KD06)
Hybomitra bimaculata (spP84843)
0.5
