# Supplementary material for: Transcriptomic Analysis of Four Cerianthid (Cnidaria, Ceriantharia) Venoms
Source: Mar Drugs. 2020 Aug 5;18(8):413. doi: 10.3390/md18080413 (PMC7460484; doi:10.3390/md18080413)
Supplement: Supplementary file 1 [file marinedrugs-18-00413-s001.zip › Individual supplementary_figs.zip!/supplementary_figs/SuppFig6_cerianthid_CLEC.pdf]

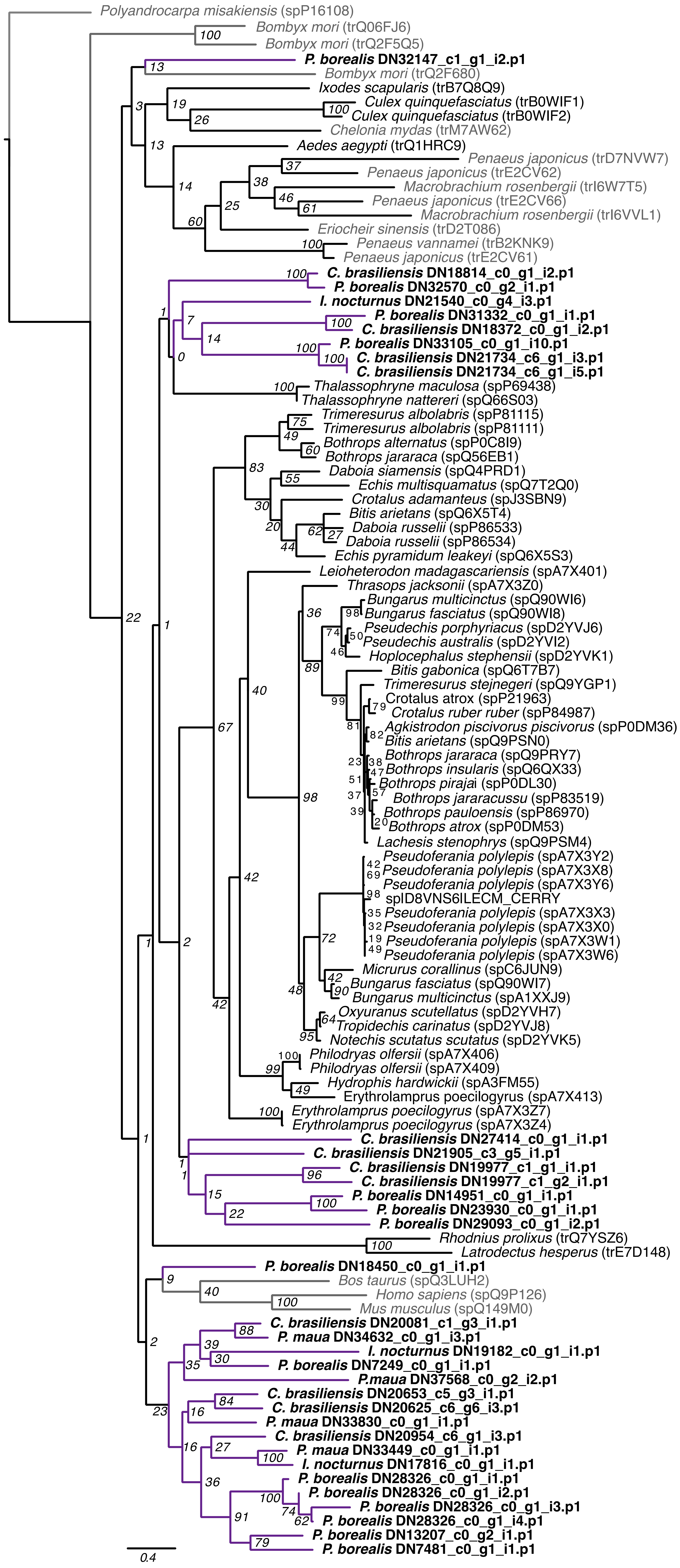

Polyandrocarpa misakiensis (spP16108)
Bombyx mori (trQ06FJ6)
Bombyx mori (trQ2F5Q5)
P. borealis DN32147_c1_g1_i2.p1
Bombyx mori (trQ2F680)
Ixodes scapularis (trB7Q8Q9)
Culex quinquefasciatus (trB0WIF1)
Culex quinquefasciatus (trB0WIF2)
Chelonia mydas (trM7AW62)
Aedes aegypti (trQ1HRC9)
Penaeus japonicus (trD7NVW7)
Penaeus japonicus (trE2CV62)
Macrobrachium rosenbergii (trI6W7T5)
Penaeus japonicus (trE2CV66)
Macrobrachium rosenbergii (trI6VVL1)
Eriocheir sinensis (trD2T086)
Penaeus vannamei (trB2KNK9)
Penaeus japonicus (trE2CV61)
C. brasiliensis DN18814_c0_g1_i2.p1
P. borealis DN32570_c0_g2_i1.p1
I. nocturnus DN21540_c0_g4_i3.p1
P. borealis DN31332_c0_g1_i1.p1
C. brasiliensis DN18372_c0_g1_i2.p1
P. borealis DN33105_c0_g1_i10.p1
C. brasiliensis DN21734_c6_g1_i3.p1
C. brasiliensis DN21734_c6_g1_i5.p1
Thalassophryne maculosa (spP69438)
Thalassophryne nattereri (spQ66S03)
Trimeresurus albolabris (spP81115)
Trimeresurus albolabris (spP81111)
Bothrops alternatus (spP0C8I9)
Bothrops jararaca (spQ56EB1)
Daboia siamensis (spQ4PRD1)
Echis multisquamatus (spQ7T2Q0)
Crotalus adamanteus (spJ3SBN9)
Bitis arietans (spQ6X5T4)
Daboia russelii (spP86533)
Daboia russelii (spP86534)
Echis pyramidum leakeyi (spQ6X5S3)
Leioheterodon madagascariensis (spA7X401)
Thrasops jacksonii (spA7X3Z0)
Bungarus multicinctus (spQ90WI6)
Bungarus fasciatus (spQ90WI8)
Pseudechis porphyriacus (spD2YVJ6)
Pseudechis australis (spD2YVI2)
Hoplocephalus stephensii (spD2YVK1)
Bitis gabonica (spQ6T7B7)
Trimeresurus stejnegeri (spQ9YGP1)
Crotalus atrox (spP21963)
Crotalus ruber ruber (spP84987)
Agkistrodon piscivorus piscivorus (spP0DM36)
Bitis arietans (spQ9PSN0)
Bothrops jararaca (spQ9PRY7)
Bothrops insularis (spQ6QX33)
Bothrops pirajai (spP0DL30)
Bothrops jararacussu (spP83519)
Bothrops pauloensis (spP86970)
Bothrops atrox (spP0DM53)
Lachesis stenophrys (spQ9PSM4)
Pseudoferania polylepis (spA7X3Y2)
Pseudoferania polylepis (spA7X3X8)
Pseudoferania polylepis (spA7X3Y6)
spID8VNS6ILECM_CERRY
Pseudoferania polylepis (spA7X3X3)
Pseudoferania polylepis (spA7X3X0)
Pseudoferania polylepis (spA7X3W1)
Pseudoferania polylepis (spA7X3W6)
Micrurus corallinus (spC6JUN9)
Bungarus fasciatus (spQ90WI7)
Bungarus multicinctus (spA1XXJ9)
Oxyuranus scutellatus (spD2YVH7)
Tropidechis carinatus (spD2YVJ8)
Notechis scutatus scutatus (spD2YVK5)
Philodryas olfersii (spA7X406)
Philodryas olfersii (spA7X409)
Hydrophis hardwickii (spA3FM55)
Erythrolamprus poecilogyrus (spA7X413)
Erythrolamprus poecilogyrus (spA7X3Z7)
Erythrolamprus poecilogyrus (spA7X3Z4)
C. brasiliensis DN27414_c0_g1_i1.p1
C. brasiliensis DN21905_c3_g5_i1.p1
C. brasiliensis DN19977_c1_g1_i1.p1
C. brasiliensis DN19977_c1_g2_i1.p1
P. borealis DN14951_c0_g1_i1.p1
P. borealis DN23930_c0_g1_i1.p1
P. borealis DN29093_c0_g1_i2.p1
Rhodnius prolixus (trQ7YSZ6)
Latrodectus hesperus (trE7D148)
P. borealis DN18450_c0_g1_i1.p1
Bos taurus (spQ3LUH2)
Homo sapiens (spQ9P126)
Mus musculus (spQ149M0)
C. brasiliensis DN20081_c1_g3_i1.p1
P. maua DN34632_c0_g1_i3.p1
I. nocturnus DN19182_c0_g1_i1.p1
P. borealis DN7249_c0_g1_i1.p1
P.maua DN37568_c0_g2_i2.p1
C. brasiliensis DN20653_c5_g3_i1.p1
C. brasiliensis DN20625_c6_g6_i3.p1
P. maua DN33830_c0_g1_i1.p1
C. brasiliensis DN20954_c6_g1_i3.p1
P. maua DN33449_c0_g1_i1.p1
I. nocturnus DN17816_c0_g1_i1.p1
P. borealis DN28326_c0_g1_i1.p1
P. borealis DN28326_c0_g1_i2.p1
P. borealis DN28326_c0_g1_i3.p1
P. borealis DN28326_c0_g1_i4.p1
P. borealis DN13207_c0_g2_i1.p1
P. borealis DN7481_c0_g1_i1.p1
0.4
