# Supplementary material for: Transcriptomic Analysis of Four Cerianthid (Cnidaria, Ceriantharia) Venoms
Source: Mar Drugs. 2020 Aug 5;18(8):413. doi: 10.3390/md18080413 (PMC7460484; doi:10.3390/md18080413)
Supplement: Supplementary file 1 [file marinedrugs-18-00413-s001.zip › Individual supplementary_figs.zip!/supplementary_figs/SuppFig7_cerianthid_cystatin.pdf]

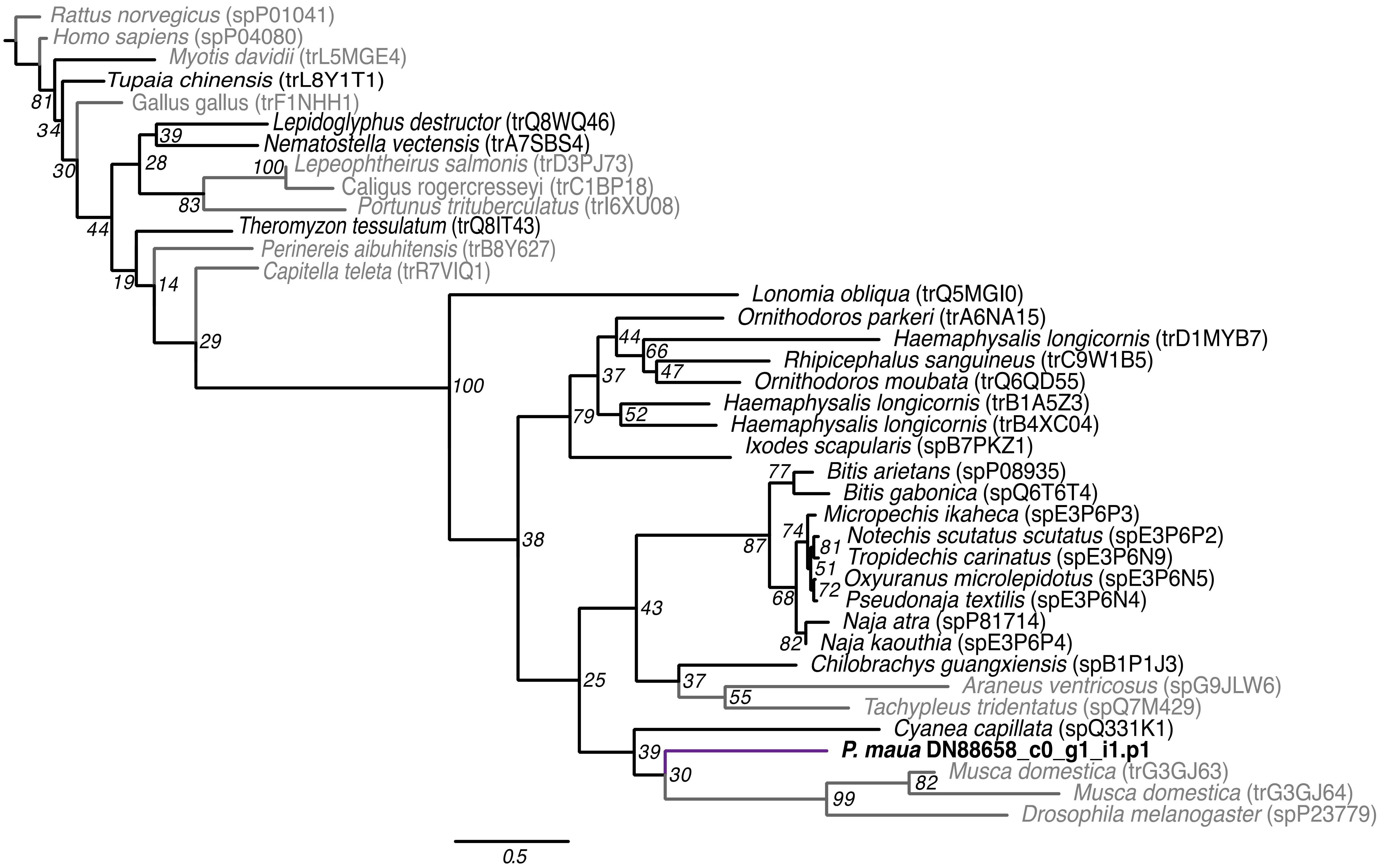
Rattus norvegicus (spP01041)
Homo sapiens (spP04080)
Myotis davidii (trL5MGE4)
Tupaia chinensis (trL8Y1T1)
Gallus gallus (trF1NHH1)
Lepidoglyphus destructor (trQ8WQ46)
Nematostella vectensis (trA7SBS4)
Lepeophtheirus salmonis (trD3PJ73)
Caligus rogercresseyi (trC1BP18)
Portunus trituberculatus (trI6XU08)
Theromyzon tessulatum (trQ8IT43)
Perinereis aibuhitensis (trB8Y627)
Capitella teleta (trR7VIQ1)
Lonomia obliqua (trQ5MGI0)
Ornithodoros parkeri (trA6NA15)
Haemaphysalis longicornis (trD1MYB7)
Rhipicephalus sanguineus (trC9W1B5)
Ornithodoros moubata (trQ6QD55)
Haemaphysalis longicornis (trB1A5Z3)
Haemaphysalis longicornis (trB4XC04)
Ixodes scapularis (spB7PKZ1)
Bitis arietans (spP08935)
Bitis gabonica (spQ6T6T4)
Micropechis ikaheca (spE3P6P3)
Notechis scutatus scutatus (spE3P6P2)
Tropidechis carinatus (spE3P6N9)
Oxyuranus microlepidotus (spE3P6N5)
Pseudonaja textilis (spE3P6N4)
Naja atra (spP81714)
Naja kaouthia (spE3P6P4)
Chilobrachys guangxiensis (spB1P1J3)
Araneus ventricosus (spG9JLW6)
Tachypleus tridentatus (spQ7M429)
Cyanea capillata (spQ331K1)
P. maua DN88658_c0_g1_i1.p1
Musca domestica (trG3GJ63)
Musca domestica (trG3GJ64)
Drosophila melanogaster (spP23779)
0.5
