# Supplementary material for: Transcriptomic Analysis of Four Cerianthid (Cnidaria, Ceriantharia) Venoms
Source: Mar Drugs. 2020 Aug 5;18(8):413. doi: 10.3390/md18080413 (PMC7460484; doi:10.3390/md18080413)
Supplement: Supplementary file 1 [file marinedrugs-18-00413-s001.zip › Individual supplementary_figs.zip!/supplementary_figs/SuppFig8_cerinathid_venom302.pdf]

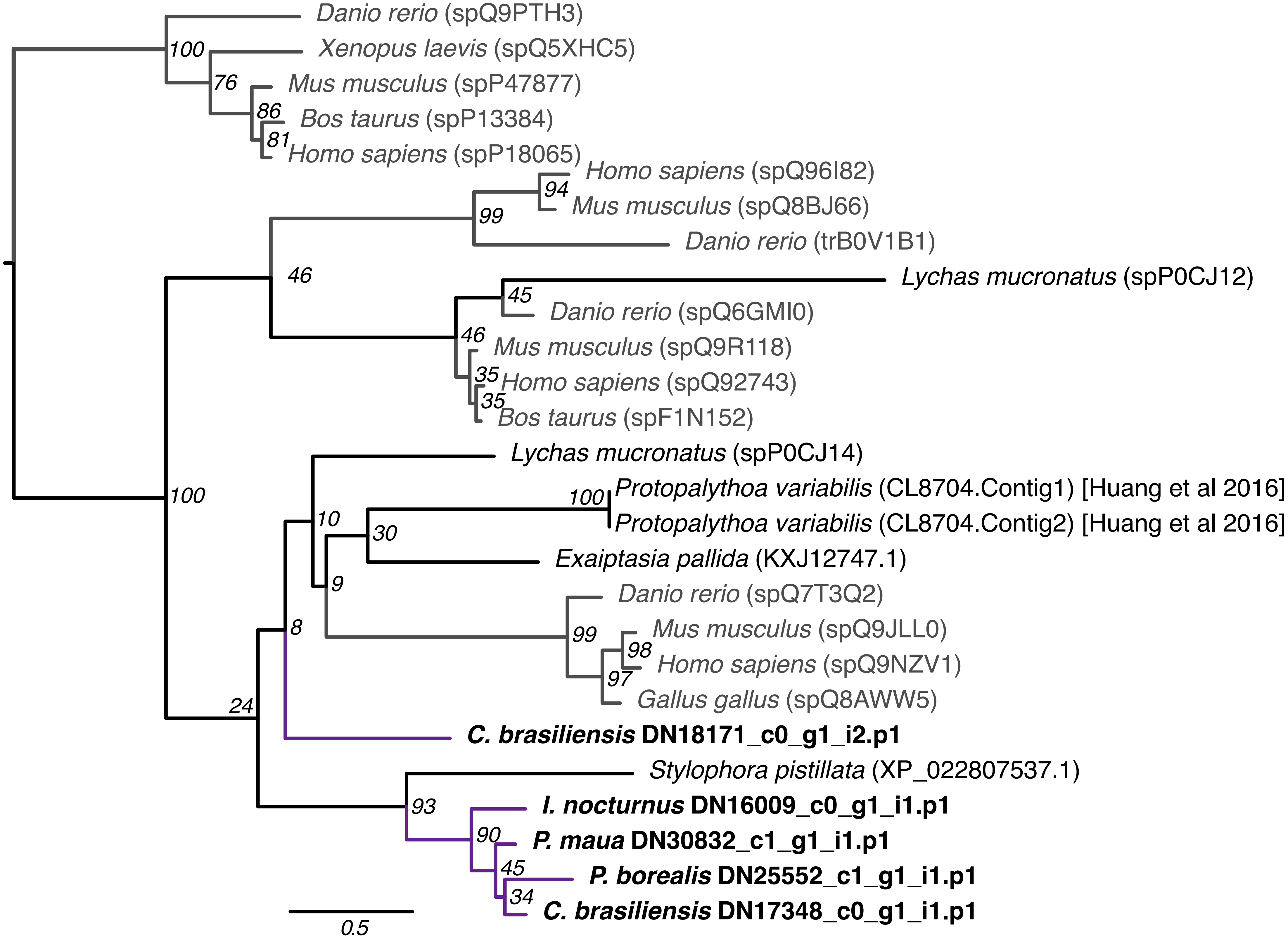

Danio rerio (spQ9PTH3)
Xenopus laevis (spQ5XHC5)
Mus musculus (spP47877)
Bos taurus (spP13384)
Homo sapiens (spP18065)
Homo sapiens (spQ96I82)
Mus musculus (spQ8BJ66)
Danio rerio (trB0V1B1)
Lychas mucronatus (spP0CJ12)
Danio rerio (spQ6GMI0)
Mus musculus (spQ9R118)
Homo sapiens (spQ92743)
Bos taurus (spF1N152)
Lychas mucronatus (spP0CJ14)
Protopalythoa variabilis (CL8704.Contig1) [Huang et al 2016]
Protopalythoa variabilis (CL8704.Contig2) [Huang et al 2016]
Exaiptasia pallida (KXJ12747.1)
Danio rerio (spQ7T3Q2)
Mus musculus (spQ9JLL0)
Homo sapiens (spQ9NZV1)
Gallus gallus (spQ8AWW5)
C. brasiliensis DN18171_c0_g1_i2.p1
Stylophora pistillata (XP_022807537.1)
I. nocturnus DN16009_c0_g1_i1.p1
P. maua DN30832_c1_g1_i1.p1
P. borealis DN25552_c1_g1_i1.p1
C. brasiliensis DN17348_c0_g1_i1.p1
100
76
86
81
94
99
46
45
46
35
35
100
100
10
30
9
99
98
97
8
24
93
90
45
34
0.5
