# Supplementary material for: Transcriptomic Analysis of Four Cerianthid (Cnidaria, Ceriantharia) Venoms
Source: Mar Drugs. 2020 Aug 5;18(8):413. doi: 10.3390/md18080413 (PMC7460484; doi:10.3390/md18080413)
Supplement: Supplementary file 1 [file marinedrugs-18-00413-s001.zip › Individual supplementary_figs.zip!/supplementary_figs/SuppFig9_cerianthid_M12.pdf]

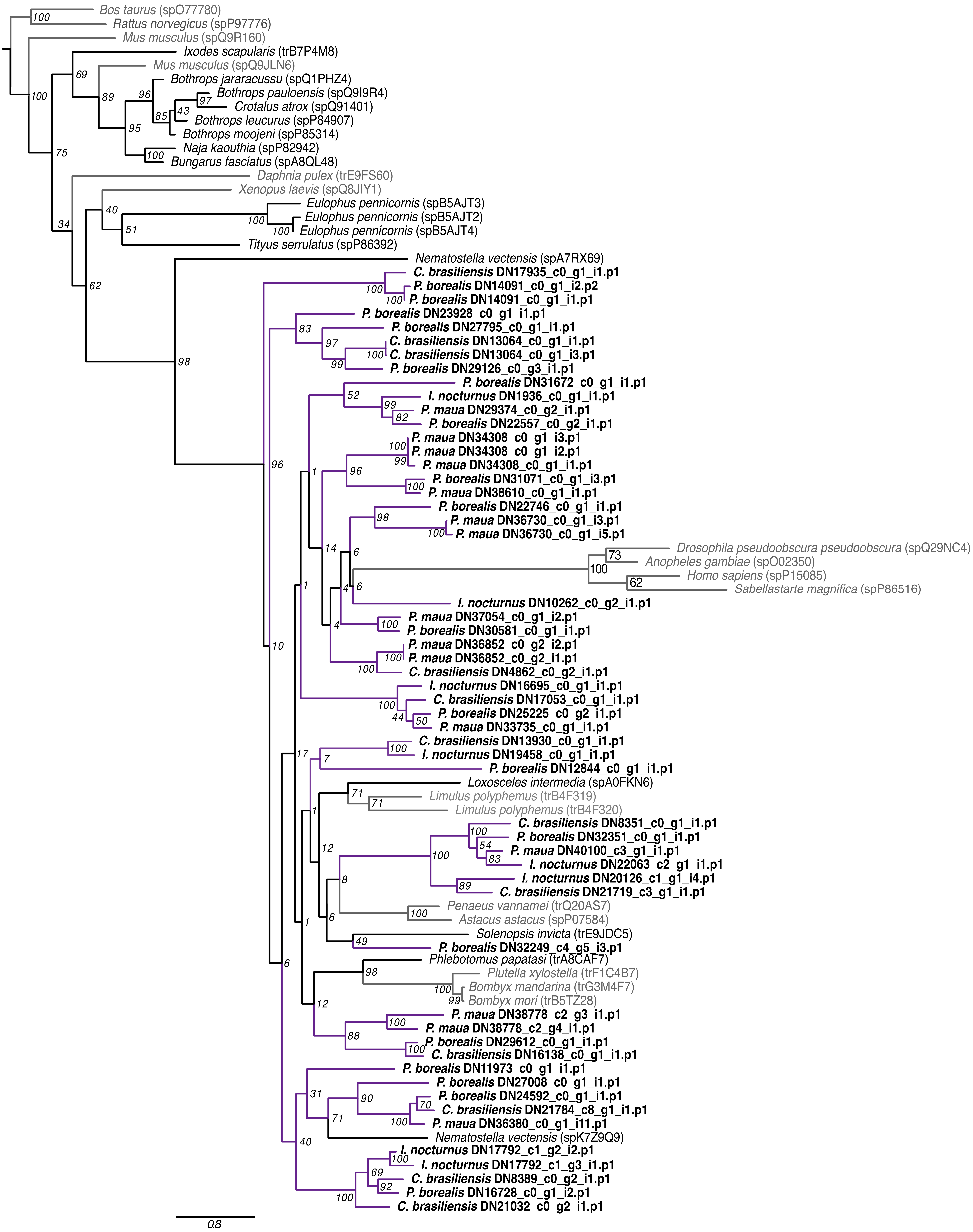

Bos taurus (spO77780)
Rattus norvegicus (spP97776)
Mus musculus (spQ9R160)
Ixodes scapularis (trB7P4M8)
Mus musculus (spQ9JLN6)
Bothrops jararacussu (spQ1PHZ4)
Bothrops pauloensis (spQ9I9R4)
Crotalus atrox (spQ91401)
Bothrops leucurus (spP84907)
Bothrops moojeni (spP85314)
Naja kaouthia (spP82942)
Bungarus fasciatus (spA8QL48)
Daphnia pulex (trE9FS60)
Xenopus laevis (spQ8JIY1)
Eulophus pennicornis (spB5AJT3)
Eulophus pennicornis (spB5AJT2)
Eulophus pennicornis (spB5AJT4)
Tityus serrulatus (spP86392)
Nematostella vectensis (spA7RX69)
C. brasiliensis DN17935_c0_g1_i1.p1
P. borealis DN14091_c0_g1_i2.p2
P. borealis DN14091_c0_g1_i1.p1
P. borealis DN23928_c0_g1_i1.p1
P. borealis DN27795_c0_g1_i1.p1
C. brasiliensis DN13064_c0_g1_i1.p1
C. brasiliensis DN13064_c0_g1_i3.p1
P. borealis DN29126_c0_g3_i1.p1
P. borealis DN31672_c0_g1_i1.p1
I. nocturnus DN1936_c0_g1_i1.p1
P. maua DN29374_c0_g2_i1.p1
P. borealis DN22557_c0_g2_i1.p1
P. maua DN34308_c0_g1_i3.p1
P. maua DN34308_c0_g1_i2.p1
P. maua DN34308_c0_g1_i1.p1
P. borealis DN31071_c0_g1_i3.p1
P. maua DN38610_c0_g1_i1.p1
P. borealis DN22746_c0_g1_i1.p1
P. maua DN36730_c0_g1_i3.p1
P. maua DN36730_c0_g1_i5.p1
Drosophila pseudoobscura pseudoobscura (spQ29NC4)
Anopheles gambiae (spO02350)
Homo sapiens (spP15085)
Sabellastarte magnifica (spP86516)
I. nocturnus DN10262_c0_g2_i1.p1
P. maua DN37054_c0_g1_i2.p1
P. borealis DN30581_c0_g1_i1.p1
P. maua DN36852_c0_g2_i2.p1
P. maua DN36852_c0_g2_i1.p1
C. brasiliensis DN4862_c0_g2_i1.p1
I. nocturnus DN16695_c0_g1_i1.p1
C. brasiliensis DN17053_c0_g1_i1.p1
P. borealis DN25225_c0_g2_i1.p1
P. maua DN33735_c0_g1_i1.p1
C. brasiliensis DN13930_c0_g1_i1.p1
I. nocturnus DN19458_c0_g1_i1.p1
P. borealis DN12844_c0_g1_i1.p1
Loxosceles intermedia (spA0FKN6)
Limulus polyphemus (trB4F319)
Limulus polyphemus (trB4F320)
C. brasiliensis DN8351_c0_g1_i1.p1
P. borealis DN32351_c0_g1_i1.p1
P. maua DN40100_c3_g1_i1.p1
I. nocturnus DN22063_c2_g1_i1.p1
I. nocturnus DN20126_c1_g1_i4.p1
C. brasiliensis DN21719_c3_g1_i1.p1
Penaeus vannamei (trQ20AS7)
Astacus astacus (spP07584)
Solenopsis invicta (trE9JDC5)
P. borealis DN32249_c4_g5_i3.p1
Phlebotomus papatasi (trA8CAF7)
Plutella xylostella (trF1C4B7)
Bombyx mandarina (trG3M4F7)
Bombyx mori (trB5TZ28)
P. maua DN38778_c2_g3_i1.p1
P. maua DN38778_c2_g4_i1.p1
P. borealis DN29612_c0_g1_i1.p1
C. brasiliensis DN16138_c0_g1_i1.p1
P. borealis DN11973_c0_g1_i1.p1
P. borealis DN27008_c0_g1_i1.p1
P. borealis DN24592_c0_g1_i1.p1
C. brasiliensis DN21784_c8_g1_i1.p1
P. maua DN36380_c0_g1_i11.p1
Nematostella vectensis (spK7Z9Q9)
I. nocturnus DN17792_c1_g2_i2.p1
I. nocturnus DN17792_c1_g3_i1.p1
C. brasiliensis DN8389_c0_g2_i1.p1
P. borealis DN16728_c0_g1_i2.p1
C. brasiliensis DN21032_c0_g2_i1.p1
0.8
